# Stigmatising Attitudes Towards Co-workers with HIV in the Workplace of a Metropolitan State, Southwestern Nigeria

**DOI:** 10.2174/1874613601711010067

**Published:** 2017-10-12

**Authors:** Idongesit Godwin Utuk, Kayode Omoniyi Osungbade, Taiwo Akinyode Obembe, David Ayobami Adewole, Victoria Oluwabunmi Oladoyin

**Affiliations:** 1 Department of Community Medicine, Faculty of Clinical Sciences, College of Medicine, University of Ibadan, Nigeria; 2 Department of Health Policy and Management, Faculty of Public Health, College of Medicine, University of Ibadan, Nigeria

**Keywords:** HIV/AIDs, Workplace, Stigma, Knowledge, Attitude, Transmission

## Abstract

**Background:**

Despite demonstrating global concerns about infection in the workplace, very little research has explored how co-workers react to those living with HIV in the workplace in sub-Saharan Africa. This study aimed to assess the level of stigmatising attitude towards co-workers living with HIV in the workplace.

**Methods:**

The study was a descriptive cross-sectional survey involving 403 respondents. They were recruited from selected companies through a multistage sampling technique. Survey was carried out using pre-tested semi-structured questionnaires. Data were analyzed using the Statistical Package for the Social Sciences to generate frequencies, cross tabulations of variables at 5% level of significance. Logistic regression model was used to determine the predictors at 95% confidence intervals.

**Results:**

Mean age of respondents was 32.9 ± 9.4 years with 86.1% being females. Overall, slightly below two-third (63.0%) had good knowledge on transmission of HIV/AIDS while 218 (54.1%) respondents had a high stigmatising attitude towards co-workers with HIV in the workplace. More female respondents (69.6%) demonstrated high stigmatising attitudes towards co-workers with HIV in the workplace (p = 0.012). Female workers were twice more likely to have high stigmatising attitudes towards co-worker with HIV [OR 2.1 (95% CI: 1.13 – 3.83)].

**Conclusion:**

Stigma towards people living with HIV/AIDs is still very persistent in different settings. Good knowledge amongst our participants about HIV/AIDs did not translate to low stigmatising attitudes among workers. Concerted efforts and trainings on the transmission of HIV/AIDs are essential to reduce stigma that is still very prevalent in workplace settings.

## 1 INTRODUCTION

The human immunodeficiency virus (HIV) infection is a major threat to the world of work. Accordingly, the International Labor Organisation (ILO) has demonstrated increasing concerns about the infection in the workplace [1]. The ILO recognized HIV as a workplace issue which should be treated like any other illness or condition in the workplace, and thus, the Organization’s emphasis on the importance of anti-discrimination campaign at work places. This is to ensure and promote decent work environment and dignity of persons including those who are infected or affected by HIV/AIDS [1]. Despite these efforts, stigma and discrimination against HIV infected employees at workplaces are not uncommon, with a consequent loss of skilled labour and a resultant decreased productivity at workplace [1, 2]. Stigma has been described in literature as a process that results from a series of five interrelated components. In this study, stigma is described in this study more with respect to the third component (in which some group doing the labelling separates ‘them’-the stigmatized group- from ‘us’) [3].

It has been reported that discrimination and stigmatization against HIV status by employers is a global problem manifesting majorly as forced disclosure of HIV serostatus, refusal to hire or promote, exclusion in the workplace and job termination [4, 5] For instance, the People Living with HIV (PLHIV) stigma index for Zambia and Kenya shows that HIV infected individuals experience significant barriers to employment, which includes discrimination in hiring, loss of promotion and job termination, because of their HIV sero-positive status [6]. In a Ugandan study, HIV infection was found to have strong levels of internal stigmatization and stigma was associated with delayed access to care, maladaptive coping and delayed disclosure to health workers and close family members or sexual partners [7].

Besides the employer, other important stakeholders in the workplace who are perpetrators of stigmatising and discriminatory attitudes towards workers living with HIV are co-workers [8, 9]. However limited research on employees as the perpetrators of this attitude exists in Nigeria [10], while more research has been carried out on employers. Furthermore, the workplace is an ideal location for information and education programmes designed to limit the spread of HIV/AIDS and to encourage proper and informed behaviour towards those who are infected with the disease [1, 11].

This study therefore assessed the level of stigmatising attitude towards co-workers with HIV in the workplace and also determined the factors associated with the stigmatising attitude.

## 2 METHODS

### 2.1 Study Setting

This cross-sectional study was carried out in Ibadan metropolis, the capital of Oyo state, employing a quantitative survey. Ibadan was the centre of administration of the old Western Region Nigeria since the days of the British colonial rule. It is situated 78 miles inland from Lagos and is a prominent transit point between the coastal region and the areas to the north. Its population is estimated to be about 3,800,000 [12]. The principal inhabitants of the city are the Yorubas. The city hosts quite a number of small, medium and large scale industries involved in the production of food and beverages, clothing and textiles, chemicals and pharmaceuticals, confectionaries, leather-works and furniture, plastics, blocks, etc.

A simple random sampling technique was employed to recruit the study participants. The industries in the study area were divided into medium and large scale industries based on a list of 14 registered industries obtained from the Manufacturing Association of Nigeria (MAN) and Ibadan Chamber of Commerce, Industries, Mines and Agriculture. From the sampling frame of each sub-group, two industries each were selected from the large scale industries and small scale industries respectively by balloting in a simple random manner. Proportionate allocation of sample size was used to determine the total number of participants selected in each industry based on the staff strength in each of the selected industries. Simple random selection of participants for survey was done using tables of random numbers (Winpepi software) generated from the staff employment list obtained from each of the industries. The generated numbers were used to identify the employees for survey.

The study population consisted of all permanent and temporary employees who had been working for at least six months in the selected industries at the time of data collection. A minimum sample size of 402 was calculated using the Leslie Kish formula for survey sampling [13]. A total 403 respondents were however surveyed.

### 2.2 Study Instrument and Data Collection

A semi-structured interviewer administered questionnaire was used to obtain data on socio-demographics characteristics, knowledge of HIV/AIDS transmission, knowledge of workplace HIV/AIDS policy and programmes and stigmatising attitudes towards co-workers with HIV in the workplace. The instrument was adapted and modified from the HIV/AIDS behaviour communication change tool kit for the workplace developed by International Labour Organization and Family Health International [14]. The questionnaire was developed in English language but was translated to the local language (Yoruba) and then back translated to English language to ensure that its original meaning was retained. To ensure data quality, the questionnaire was pretested on a similar population, outside the study area. Four research assistants were trained and were involved in data collection for the study.

## 3 Data Analysis

The questionnaires were checked daily for consistency and completeness and were coded before entry into the computer. Data was managed using the Statistical Package for the Social Sciences (SPSS) version 22 software. The independent variables of interest were summarized using frequencies, proportions, mean, medians and standard deviation.

The dependent variable was “*stigmatising attitude towards co-worker with HIV”*. It was assessesed based on a 5-point Likert scale comprising of five items. Responses to each statement was scored from 1 (strongly agree) to 5 (strongly disagree). The minimum obtainable score was 5 and the maximum obtainable score was 25. Using the median score of 12 as cut off, the stigmatising attitude towards co-worker with HIV was dichotomized into Low/Positive and High/Negative stigmatising attitudes. Respondents with scores below the median score were categorized as having a low stigmatising attitude towards co-workers with HIV (*i.e.* a positive attitude towards co-workers with HIV) while those with the median score and above were categorized as having a high stigmatising attitude (*i.e.* a negative attitude towards co-workers with HIV).

Knowledge on HIV transmission was computed from five questions while knowledge of HIV/AIDS workplace policy was computed from eight questions. Response to each question ranged from “Yes”, “No”, to “Don’t know”. Each correct answer was scored 1 and the wrong answers were scored 0. The minimum and maximum obtainable scores were 0 and 5 respectively for the knowledge on HIV transmission and 0 and 8 respectively for the knowledge of HIV/AIDS workplace policy. Scores equal to or greater than the median score of 4 were categorized as good knowledge of HIV transmission while scores less than 4 were considered poor knowledge of HIV transmission. Similarly, knowledge of HIV/AIDS workplace policy was dichotomized based on the median score. Scores equal to or greater than 7 were considered as adequate knowledge of HIV/AIDS workplace policy while scores less than 7 were considered as inadequate knowledge of HIV/AIDS workplace policy.

Bivariate analysis was carried out to determine the association between the dependent and independent variables of interest at 5% level of statistical significance. Subsequently, all variables that were significant at the 10% level were then selected and fit into the logistic regression model to determine the predictors of high/negative stigmatising attitude towards co-worker with HIV.

## 4 Ethical Consideration

Ethical approval for the study was obtained from the UI/UCH Ethics Review Committee. Permission and approval to conduct the study was also obtained from the management of the selected industries. In addition, the purpose of the study was explained to the participants and their written and verbal consents obtainted before the questionnaires were administered.

## 5 RESULTS

### 5.1 Socio-demographic Characteristics

The mean age of the respondents was 32.9 ± 9.4 years. Most (86.1%) of the respondents were females. A little above half were married (52.0%) and had tertiary education as their highest level of education (54.3%). Close to half (49.6%) have been working with their companies for more than three years (Table **1**).

### 5.2 Knowledge of HIV/AIDS

Quite a number of the people surveyed had correct responses to the knowledge question on HIV/AIDS transmission. Over four-fifth (82.1%) correctly knew that staying faithful to one’s partner will reduce the risk of contracting HIV, 78.4% correctly knew that the use of condoms during sexual intercourse will reduce a worker’s chance of contracting HIV, 71.7% correctly knew that HIV/AIDS cannot be transmitted via mosquito bites. Overall, slightly below two-third (63.0%) had good knowledge on transmission of HIV/AIDS (Table **2**).

Majority of the respondents were knowledgeable about the various aspects of the HIV/AIDS policy inquired. Three hundred and fifty-eight (88.8%) correctly knew that attending voluntary counselling and HIV testing is in workers interest to stay healthy, 82.1% correctly knew that there should be no discrimination in the workplace against people living HIV/AIDS, 76.2% correctly knew that there should be no denial of employment based on HIV status, 75.2% correctly knew that there should be no form of disclosure of a worker’s HIV status without consent and about two-thirds (68.7%) correctly knew that workers should be protected from mandatory HIV testing for employment or promotion. Overall, a considerable number (62.0%) of the respondents had adequate knowledge on HIV/AIDS workplace policy (Table **3**).

### 5.3 Stigmatising Attitudes Towards Co-workers with HIV in the Workplace

Slightly over 40% of respondents in this study strongly agreed that they will be willing to work alongside a co-worker who is HIV positive, 32.8% strongly agreed that they will be willing to eat food at company canteen prepared by a co-worker who is HIV positive and 37.2% agreed that they will be willing to use the same toilet with a co-worker who is HIV positive. About a quarter (27.8%) strongly disagreed that they will be willing to share utensils with a co-worker who is HIV positive (Table **4**). Overall, 218 (54.1%) respondents had a high stigmatising attitude towards co-workers with HIV in the workplace.

Sex of the respondent was the only socio-demographic characteristics significantly associated with stigmatising attitudes towards co-worker with HIV in the workplace. A higher proportion of female respondents (69.6%) had high stigmatising attitudes towards co-workers with HIV in the workplace compared to 51.6% respondents who were males (p = 0.012). Age, religion, marital status, ethnicity, level of education, type of industry and length of time in the company were not associated with stigmatising attitude towards co-worker with HIV in the workplace (Table **5**).

Although not statistically significant, more respondents with good knowledge about HIV/AIDS transmission (57.5%) had high stigmatising attitudes towards co-workers with HIV compared to those with poor knowledge (p = 0.075) surprisingly. Knowledge about HIV workplace policy was also not statistically associated with stigmatising attitudes towards co-worker with HIV in the workplace (p = 0.961) (Table **5**).

The only predictor of high stigmatising attitudes towards co-worker with HIV was sex. Female workers were twice more likely to have a high stigmatising attitudes towards co-worker with HIV [OR 2.1 (CI: 1.129 – 3.825)] (Table **5**).

## 6 DISCUSSION

The present paper examined the level of stigmatising attitudes towards co-workers with HIV in the workplace as well as the factors associated with the stigmatising attitudes. The results of this study showed that a high level of stigmatising attitudes towards co-workers with HIV in the workplace exists and female workers were found to be more likely to engage in this.

The above half prevalence of workers that stigmatize against PLHIV mimics findings in a national survey in which about 50% of the population were in agreement that PLHIV should be ashamed of themselves and should be blamed for bringing the disease to the community [15] Notwithstanding, the finding of a high level of stigmatising attitude towards co-workers with HIV in the workplace in this study differs from some published studies [16, 17]. However, it is in agreement with similar previous studies in South Africa and Pakistan [8, 9]. Another remarkable finding from this study was that the respondents were generally knowledgeable about the transmission of HIV and this is consistent with the findings of Pirie and Coetsee in South Africa [18]. However, the combination of the findings on knowledge and attitude in the current study suggests that a knowledge-attitude gap exists and this calls for concern as this may weaken efforts towards HIV prevention and treatment services as has been previously documented. So at every opportunity, intervention programs aimed at prevention and treatment of HIV/AIDS in the workplace should reiterate correct and complete information comprising of knowledge about the infection and attitudes towards those infected or affected by the infection. Anti-discrimination legislation as articulated by Campbell and colleagues can be employed in the reduction of stigma within the workplace amongst employees living with HIV/AIDs [5] considering the limitations of information based awareness programs and community participation as alternative strategies.

In this study, stigmatising attitudes were exhibited more by female workers. This is in contrast to Adeyemo and colleague in Nigeria [10] and Tee and colleague in Malaysia [17] who found no correlation between sex and stigmatising attitudes. Nevertheless, it mirrors findings in some of the correlates examined by Dahlui *et al* (2015) that found female poorer than men in terms of stigma in the market place [15]. The fear of contracting HIV is a possible explanation why stigmatising attitudes towards co-workers with HIV was more among women in this study. Studies have shown that women are more concerned about health and therefore exhibit better health seeking behavior [19]. Too much concern about this may however lead to inappropriate level precautionary measures including ‘been too careful’ and thus stigmatising attitudes towards HIV positive individuals especially at workplace. This explanation was corroborated by Adeyemo and colleague [10] in their study on the pre-dispositional factors in stigmatization and discrimination against HIV/AIDS seropositive persons in the workplace which found that being a female had a significant correlation with fear of HIV contagion. Intervention programs aimed at prevention and treatment of HIV in the workplace should therefore pay special attention on female workers. This step may be helpful in reducing stigmatising attitudes among them. The poor translation of adequate knowledge with stigmatizing attitudes emphasizes the need for innovative interventions that can significantly promote stigma reduction in the workplace. Culturally informed strategies [20] supported by legal safeguards in form of antidiscrimination legislation [5, 21] offer better promises for stigma reduction in the workplace within the country’s social context over awareness programs. Exploration and integration of improved provision of highly active anti-retroviral therapy (HAART) in the health clinics of industries is also an additional policy initiative that can be explored to reduce the level of stigma and discrimination at these workplaces as documented in literature [7].

This study ought to be interpreted bearing the following limitations in mind. Just as with cross-sectional surveys, our study can only determine associated factors but it is not able to establish causation. It will be desirable to explore and probe reasons behind stigma in the workplace despite adequate knowledge of the HIV/AIDs. Qualitative studies and triangulation of findings should be undertaken in future studies to establish a deeper understanding of the factors influencing stigma reduction amongst co-workers of PLHIV.

## CONCLUSION

This study has shown that stigmatising attitudes towards co-workers with HIV in the workplace still exist particularly among female workers. Workplace policies and anti-discrimination legislature aimed at addressing HIV/AIDS, with special focus on female workers, can help reduce the stigma of the disease. This will also serve to create a positive environment where people living with, or affected by HIV/AIDS would be more productive and therefore contribute to the growth and development of the society.

## Figures and Tables

**Table 1:** Socio-demographic characteristics of respondents.

**Variables**	**Frequency** **(N =403)**	**Percentage** **(%)**
**Age (in years)** ­ ­ ­ ­ < 25 ­ ­ ­ ­ 25 – 34 ­ ­ ­ ­ 35 – 44 ­ ­ ­ ­ ≥ 45	74 168 95 58	18.7 42.5 24.1 14.7
Age: Mean ± SD*	32.9 ± 9.4 years	
**Sex** ­ ­ ­ ­ Male ­ ­ ­ ­ Female	347 56	86.1 13.9
**Religion** ­ ­ ­ ­ Christianity ­ ­ ­ ­ Islam ­ ­ ­ ­ Traditional	292 110 1	72.5 27.3 0.2
**Marital status** ­ ­ ­ ­ Single ­ ­ ­ ­ Married ­ ­ ­ ­ Widowed ­ ­ ­ ­ Cohabiting	188 209 4 1	46.8 52.0 1.0 0.2
**Ethnicity** ­ ­ ­ ­ Yoruba ­ ­ ­ ­ Igbo ­ ­ ­ ­ Hausa ­ ­ ­ ­ Others	302 53 13 31	75.7 13.3 3.3 7.8
**Highest educational level** ­ ­ ­ ­ No formal education ­ ­ ­ ­ Primary ­ ­ ­ ­ Secondary ­ ­ ­ ­ Tertiary ­ ­ ­ ­ Others	7 18 149 213 5	1.8 4.6 38.1 54.3 1.3
**Type of Industry** ­ ­ ­ ­ Medium scale ­ ­ ­ ­ Large scale	170 233	42.2 57.8
**Length of time in company (in years)** ­ ­ ­ ­ 6 months - 1 ­ ­ ­ ­ ˃1 year - 3 ­ ­ ­ ­ Above 3	97 100 194	24.8 25.6 49.6

*SD – Standard Deviation

**Table 2:** Respondents’ knowledge on HIV/AIDS transmission.

**Variables**	**Frequency** **(N =403)**	**Percentage** **(%)**
**Faithfulness to partner will reduce risk of HIV infection** ­ ­ ­ ­ Yes ­ ­ ­ ­ No ­ ­ ­ ­ Don’t Know	331 50 22	82.1 12.4 5.4
**A healthy looking person could be HIV positive** ­ ­ ­ ­ Yes ­ ­ ­ ­ No ­ ­ ­ ­ Don’t Know	329 56 18	81.6 13.9 4.4
**Use of condom during sexual intercourse reduces risk of HIV infection** ­ ­ ­ ­ Yes ­ ­ ­ ­ No ­ ­ ­ ­ Don’t Know	316 60 27	78.4 14.9 6.7
**HIV can be transmitted by supernatural means** ­ ­ ­ ­ Yes ­ ­ ­ ­ No ­ ­ ­ ­ Don’t Know	93 242 68	23.1 60.0 16.2
**HIV can be transmitted by mosquito bites** ­ ­ ­ ­ Yes ­ ­ ­ ­ No ­ ­ ­ ­ Don’t Know	76 289 38	18.9 71.7 9.4
**Overall knowledge of HIV/AIDS transmission** ­ ­ ­ ­ Good ­ ­ ­ ­ Poor	254 149	63.0 37.0

**Table 3:** Respondents’ knowledge of HIV/AIDS workplace policy.

**Variables**	**Frequency** **(N =403)**	**Percentage** **(%)**
**Workers uptake of VCT will help to stay healthy** ­ ­ ­ ­ Yes ­ ­ ­ ­ No ­ ­ ­ ­ Don’t Know	358 22 23	88.8 5.5 5.7
**Support workers right to workplace HIV education** ­ ­ ­ ­ Yes ­ ­ ­ ­ No ­ ­ ­ ­ Don’t Know	348 29 26	86.4 7.2 6.5
**Support no discrimination policy against PLHIV at workplace** ­ ­ ­ ­ Yes ­ ­ ­ ­ No ­ ­ ­ ­ Don’t Know	331 47 25	82.1 11.7 6.2
**Support dialogue between management and workers on workplace HIV/AIDS policy** ­ ­ ­ ­ Yes ­ ­ ­ ­ No ­ ­ ­ ­ Don’t Know	321 40 42	79.7 9.9 10.4
**Support no termination of job if fit to work** ­ ­ ­ ­ Yes ­ ­ ­ ­ No ­ ­ ­ ­ Don’t Know	319 49 36	79.2 11.9 8.9
**Support no denial of employment if based on HIV status** ­ ­ ­ ­ Yes ­ ­ ­ ­ No ­ ­ ­ ­ Don’t Know	307 58 38	76.2 14.4 9.4
**Support confidentiality about HIV status** ­ ­ ­ ­ Yes ­ ­ ­ ­ No ­ ­ ­ ­ Don’t Know	303 66 34	75.2 16.4 8.4
**Support no mandatory HIV testing for employment or promotion** ­ ­ ­ ­ Yes ­ ­ ­ ­ No ­ ­ ­ ­ Don’t Know	277 95 31	68.7 23.6 7.7
**Overall knowledge of HIV/AIDS policy** ­ ­ ­ ­ Adequate ­ ­ ­ ­ Inadequate	250 153	62.0 38.0

**Table 4:** Respondents’ stigmatising attitudes towards co-worker with HIV.

Variables	SA* n (%)	A* n (%)	UD* n (%)	D* n (%)	SD* n (%)
Willing to work with a HIV+ co-worker	175 (43.4)	145(36.0)	31 (7.7)	21(5.2)	31(7.7)
Willing to eat food prepared by a HIV+ co-worker	132 (32.8)	122((30.3)	46(11.4)	31(7.7)	72(17.9)
Willing to share utensils with a HIV+ co-worker	82(20.3)	108(26.8)	41(10.2)	60(14.9)	112(27.8)
Willing to use the same toilet with a HIV+ co-worker	101(25.1)	150(37.2)	36(8.9)	44(10.9)	72(17.9)
Willing to receive medical treatment from a health-care co-worker who is HIV-positive	109(27.0)	96(23.8)	46(11.4)	72(17.9)	80(19.9)

*SA - Strongly agree, A – Agree, UD – Undecided, D – Disagree, SD – Strongly disagree

**Table 5:** Association between stigmatising attitude, knowledge of HIV/AIDS transmission, knowledge of workplace policy on HIV/AIDS and selected socio-demographic characteristics.

**Variables**	**Stigmatising attitude**		**P-value^a^**	**Adjusted** **OR** **(95% CI)^b^**	**P-value^c^**
	**Low** **(%)**	**High** **(%)**			
**Age (years)** ­ ­ ­ ­ < 25 ­ ­ ­ ­ 25 – 34 ­ ­ ­ ­ 35 – 44 ­ ­ ­ ­ ≥ 45	39(52.7) 71(42.3) 43(45.3) 29(50.0)	35(47.3) 97(57.7) 52(54.7) 29(50.0)	0.444		
**Sex** ­ ­ ­ ­ Male ­ ­ ­ ­ Female	168(48.4) 17(30.4)	179(51.6) 39(69.6)	**0.012**	1 2.078(1.129-3.825)	**0.019**
**Religion** ­ ­ ­ ­ Christianity ­ ­ ­ ­ Others	132(45.2) 53(47.7)	160(54.8) 58(52.3)	0.647		
**Marital status** ­ ­ ­ ­ Currently married ­ ­ ­ ­ Not currently married	95(45.5) 90(46.6)	114(54.5) 103(53.4)	0.813		
**Ethnicity** ­ ­ ­ ­ Yoruba ­ ­ ­ ­ Others	145 (48.0) 40 (41.2)	157 (52.0) 57 (58.8)	0.244		
**Highest educational level** ­ ­ ­ ­ None/Primary ­ ­ ­ ­ Secondary ­ ­ ­ ­ Tertiary	11(44.0) 67(45.0) 100(46.9)	14(56.0) 82(55.0) 113(53.1)	0.913		
**Type of Industry** ­ ­ ­ ­ Medium scale ­ ­ ­ ­ Large scale	80(47.1) 105(45.1)	90(52.9) 128(54.9)	0.692		
**Length of time in company** ­ ­ ­ ­ 6 month - 1 year ­ ­ ­ ­ >1 -3 years ­ ­ ­ ­ Above 3 years	46(47.4) 43(43.0) 87(44.8)	51(52.6) 57(57.0) 107(55.2)	0.821		
**Knowledge of HIV/AIDS transmission** ­ ­ ­ ­ Good ­ ­ ­ ­ Poor	108(42.5) 77(51.7)	146(57.5) 72(48.3)	0.075	1.392(0.924-2.097) 1	0.114
**Knowledge about HIV/AIDS workplace policy** ­ ­ ­ ­ Adequate ­ ­ ­ ­ Inadequate	115(46.0) 70(45.8)	135(54.0) 83(54.2)	0.961		

^a^P-value in bivariate analysis ^b^Adjusted odds ratio (95% confidence interval); Conducted for significant values on bivariate analysis only. ^c^P-value in logistic regression
